# Prevalence and determinants of tobacco use among young people in The Gambia

**DOI:** 10.1136/bmjgh-2017-000482

**Published:** 2017-12-28

**Authors:** Isatou K Jallow, John Britton, Tessa Langley

**Affiliations:** 1 Division of Epidemiology and Public Health, UK Center for Tobacco and Alcohol Studies, University of Nottingham, Clinical Science Building, CityHospital, Nottingham NG5 1PB, UK; 2 Ministry of Health & Social Welfare, National Public Health Laboratory, Banjul, The Gambia

**Keywords:** epidemiology, public health, cross-sectional survey

## Abstract

**Introduction:**

Tobacco consumption and consequent morbidity and mortality are expected to grow most markedly over coming decades in low-income and middle-income countries (LMICs). Preventing tobacco experimentation and uptake among young people in LMICs is therefore vital. However, data on smoking in these countries, particularly in sub-Saharan Africa, remain sparse.

**Method:**

We used two-stage cluster random sampling to select students in upper and senior secondary schools throughout The Gambia, and a self-administered questionnaire to collect data on their tobacco use, risk factors and demographic details.

**Results:**

Of 10 392 eligible students, 10 289 (99%; 55% girls and 44% boys, age 12–20 years) participated. The prevalence of ever smoking cigarettes, cigars or pipes was 16.7% (25.7% boys and 9.4% girls) and current (past 30 days) smoking 4.5% (7.9% boys and 1.5% girls). Smoking was more common among students attending private schools (OR 1.69, 95% CI 1.29 to 2.22), of Christian or other faiths compared with Muslims, living with parents (OR 1.39, 95% CI 1.06 to 1.81), who had smoking allowed in their homes (OR 1.67, 95% CI 1.30 to 2.13), with family members who smoked or had one or more friends who smoked. Most (55.6%) smokers want to stop, but only 22% received any stop smoking support. Ever smoking of shisha, at 8.1%, was unexpectedly high, and relatively prevalent among girls (11.4% of boys and 5.4% of girls).

**Conclusions:**

Tobacco use is common among young people in The Gambia. Shisha smoking is also common in this population, and in relative terms especially among girls. Further work is required to determine whether this is a problem local to The Gambia or reflects a wider pattern of tobacco use in sub-Saharan Africa.

**Key questions**

**What is already known about this topic?**

- Tobacco consumption and in due course tobacco-related morbidity and mortality are likely to grow most markedly over the coming decades in low-income and middle-income countries.
- Studies in rich countries show that most smokers begin smoking before age 18, and that between 33% and 50% of those who try smoking even few cigarettes become regular smokers.
- Since about 40% of Gambians are currently aged ≤15 years, this indicates that the number of young smokers in The Gambia is likely to rise in coming decades. However, data on young people smoking are sparse.

**What are the new findings?**

- This study provides the first comprehensively representative data on prevalence and determinants of tobacco use among young people in The Gambia.
- The findings suggest that smoking experimentation is common, particularly among boys, those attending private schools and those who are not Muslim, and that shisha smoking may be under-recognised, particularly among girls.

**Recommendation for policy**

- These findings provide valuable information for tobacco control policy and evidence to enable targeted intervention for young people most at risk.
- The results also suggest that tobacco control measures should include regular monitoring of uptake of all forms of tobacco products and not to be limited on cigarettes alone.

## Introduction

Tobacco smoking kills about 6 million people annually and is a growing global public health problem. The WHO estimates that around 1 billion people are current smokers,^1^ 80% of whom live in low-income and middle-income countries (LMICs),^2 3^ and that this total will rise to 1.7 billion by 2025.^1^ Studies in rich countries show that most smokers begin smoking before age 18,^4^ and that between 33% and 50% of those who try smoking even few cigarettes become regular smokers.^5^ There is also concern over the emergence of waterpipe (shisha) smoking among young people.^6 7^ Preventing smoking experimentation and uptake among young people in LMICs is a clear public health priority.

Since smoking prevalence is now falling in many rich countries, tobacco companies are turning to LMICs to generate new growth in tobacco sales. Tobacco consumption and in due course tobacco-related mortality are therefore likely to grow most markedly over the coming decades in LMICs.^3 8^ However, data on smoking prevalence among young people in the world’s poorest nations, particularly in sub-Saharan Africa, remain sparse.

The Gambia is a West African country of 1.9 million people with a *per capita* gross domestic product of US$471 in 2015.^9^ Although The Gambia has no tobacco industry presence, smoking prevalence among adult men, estimated at 24% (and 0.8% in women) in 2012, is common compared with many LMICs.^10^ Data on tobacco use in young people are limited however, to a study of schools in the Greater Banjul Area in 2008, in which 9% of girls and 13% of boys aged 13–15 years had smoked in the past 30 days.^11^ Since about 40% of Gambians are currently aged 15 years, this indicates that the number of young smokers in The Gambia is likely to rise in coming decades.^12 13^ To obtain a reliable and nationally representative estimate of smoking prevalence, and the major risk factors for smoking among young people in The Gambia, we conducted a survey of smoking prevalence and determinants in a national sample of Gambian schools.

## Methods

### Study population

The Gambia has a 6-3-3 education system, comprising 6 years of lower basic (grades 1–6), 3 years of upper basic (grades 7–9) and 3 years of senior secondary (grades 10–12). Children enter grade 1 at age 7. Annual progression through the grades is not automatic, and some students repeat grades. This study was carried out in Upper Basic Schools (UBS) and Senior Secondary Schools (SSS) in all six Regional Education Directorates in The Gambia. Representative samples of students in grades 7–12 were generated by two-stage cluster sampling. In the first stage, schools were randomly selected from a list of schools provided by the Ministry of Basic and Secondary Education with a probability proportional to their enrolment size. In the second stage, classes within the schools were randomly selected from the total number of classes in the schools. All students in the selected classes were eligible to participate. Our study was powered to estimate youth smoking prevalence of 15% with 1% precision, which required a minimum sample size of 4885 (Epi Info 7). Student participation was voluntary and anonymous.

### Data collection and study variables

Participating students completed a self-administered questionnaire collecting data on a range of variables including demographic details, tobacco-use indicators (including current cigarette, cigar or pipe smoking, number of days smoked during the past 30 days and age at onset of smoking); family member smoking; number of friends smoking; brand of tobacco usually smoked and price paid; usual place of purchase; use of other smoked or smokeless tobacco; and desire and support to quit smoking. The questionnaire also included a series of questions covering smoking susceptibility, exposure to secondhand smoke, support for smoking regulations, banning public smoking, exposure to tobacco advertisements and promotion, antismoking media messages, beliefs about the danger of smoking and perceived benefits of smoking: these data will be reported in a separate publication. The questionnaire was piloted in a sample of students in one of the selected schools and as a result of feedback from participants reporting shisha use, we added brief questions on ever use of shisha to the final questionnaire. The school surveys were carried out between June and December 2016.

### Statistical analysis

Ever smoking was defined as any smoking of cigarettes, cigars or pipes at any time in the past, and current smoking as use in the past 30 days. Data were entered into Microsoft Access and exported into Stata V.14 for analysis. Proportions and 95% CIs were obtained as estimates of prevalence; univariate logistic regression was first carried out to look for associations between the outcome variable (current smoking) and the exposure variables. We then adjusted associations for *a priori* confounders comprising age, gender and rural/urban area of school, and used stepwise (forward) multivariate analyses to ascertain the predicting factors of current smoking and ever shisha smoking.

## Results

### Characteristics of the study population

Our sample comprised 50 schools, of which 33 were UBS and 17 SSS; 13 were private, 27 public and 10 grant-aided. The head teachers of all sampled schools agreed to participate in the study. Our second-stage sample identified 210 classes for the survey, which according to school registers included 10 395 students. A total of 10 289 students (99%) in these classes completed the survey.

The main characteristics of study participants are described in table 1. There were more female (55.6%) than male (44.4%) participants; 63.9% were aged between 14 and 17 years and 93.1% were of Muslim faith. Most participants lived with their parents (80.2%) in homes where smoking was not allowed (70.9%), and had no family members (71.6%) or friends (66.5%) who smoked. The majority (54.1%) reported having <D15 (approximately $0.40) daily spending money (table 1).

**Table 1:** Sociodemographic characteristics of study participants

Characteristics	Categories	(n=10 289)	(%)
Gender	Boys	4567	44.3
	Girls	5722	55.6
Age (years)	12–13	960	9.3
	14– 15	2776	26.9
	16– 17	3812	37.0
	18– 19	2221	21.5
	20	525	5.1
Class	Grade 7	1507	14.6
	Grade 8	2041	19.8
	Grade 9	2215	21.5
	Grade 10	1509	14.6
	Grade 11	1592	15.4
	Grade 12	1425	13.8
School type	UBS	5785	56.2
	SSS	4504	43.7
School ownership	Public	7678	74.6
	Grant-aided	1052	10.5
	Private	1559	15.1
Religion	Muslim	9564	93.1
	Christian	602	5.8
	Other	103	1.0
Daily school money	Do not have spending money	1435	13.9
	<D15	4131	40.2
	D16−D35	3205	31.2
	>D35	1497	14.5
Living with parents	Yes	8250	80.2
	No	2029	19.7
Parents working	Father/stepfather/male guardian only	3343	32.5
	Mother/stepmother/female guardian only	1507	14.6
	Both	4300	41.8
	Neither	737	7.1
	Do not know	387	3.7
Home smoking allowed	No	7295	70.9
	Sometimes	1085	10.5
	Yes	1906	18.5
Family members who smoke	None	7364	71.6
	Mother	274	2.6
	Father	1199	11.6
	Brother/Sister	718	6.9
	Others	729	7.0
Number of friends who smoke	None	6790	66.0
	1	673	6.5
	2	356	3.4
	≥3	762	7.4
	Not sure	1699	16.5

SSS, Senior Secondary Schools; UBS, Upper Basic Schools.

### Prevalence and use of tobacco products

One in six participants (16.7%) had ever smoked tobacco, comprising around 1 in 4 (25.7%) boys and 1 in 10 (9.4%) girls and 7.9% of boys and 1.5% of girls had done so in the last 30 days (table 2). Manufactured cigarettes were the most widely used of these products (9.8% ever use; hand-rolled cigarettes 2.7%, cigars 2.3% and pipes 2.1%). After manufactured cigarettes, however, shisha was the next most widely used product, and with ever use reported by 11.4% and 5.4%, respectively, of boys and of girls, was relatively widely used by girls. Ever use of smokeless tobacco was reported by 2.7% and current use by 1.2% of participants (table 2).

**Table 2:** Prevalence tobacco use and type of tobacco product smoke by gender

Characteristics	Total n=10 289	%	95**%** CI	Boys n=4567 n (%)	Girls n=5722 n (%)
Smoking status					
Never smokers	8568	83.2	82.50 to 83.91	3389 (74.2)	5179 (90.5)
Ever smokers	1719	16.7	16.01 to 17.44	1177 (25.7)	542 (9.4)
Ex-smokers	1264	12.2	11.6 to 12.93	813 (17.8)	451 (7.8)
current smokers	455	4.4	4.04 to 4.83	364 (7.9)	91 (1.5)
Tobacco products used by ever smokers					
Cigarettes	1009	9.8	9.24 to 10.39	824 (18.0)	183 (3.1)
Hand-rolled cigarettes	279	2.7	2.41 to 3.04	225 (4.9)	54 (0.9)
Pipes	221	2.1	1.88 to 2.44	168 (3.6)	53 (0.9)
Cigar	239	2.3	2.04 to 2.63	185 (4.1)	54 (0.9)
Shisha	834	8.1	7.59 to 8.65	523 (11.4)	311 (5.4)
Smokeless tobacco					
Never users	9991	97.2	96.9 to 97.5	4350 (95.4)	5641 (98.6)
Ever users	284	2.76	2.24 to 3.0	204 (3.7)	80 (1.3)
Current users	129	1.25	1.05 to 1.28	95 (2.1)	34 (0.5)

### Ever and current smoking, and ever use of shisha, by age

**Figure 1:**
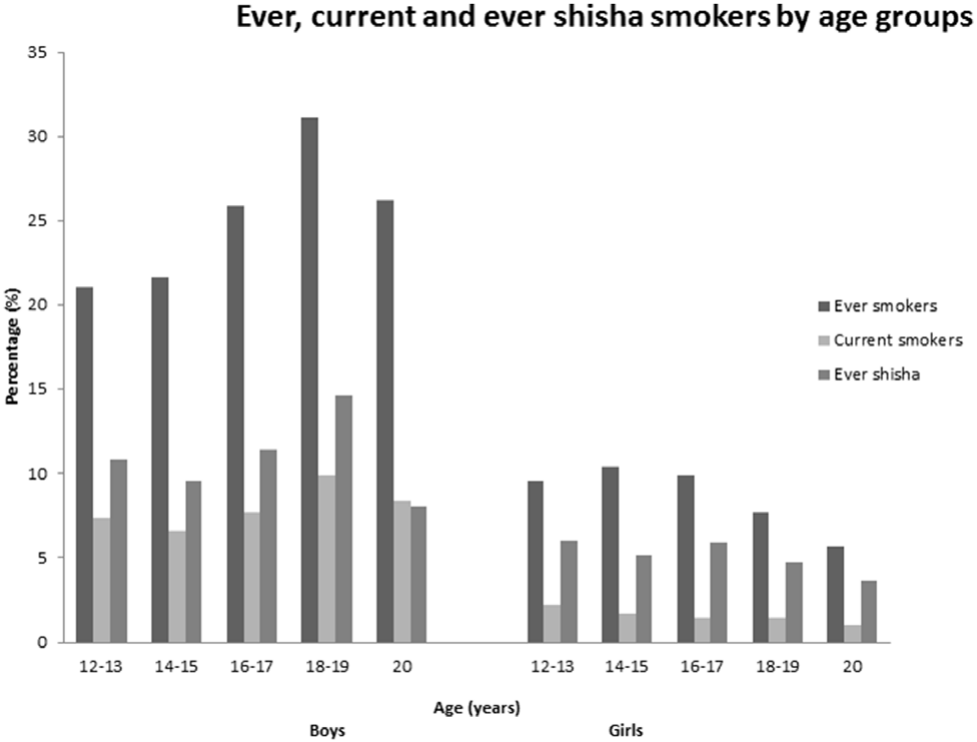
Age groups (years) of ever smokers, current smokers and ever shisha smokers.

The proportion of participants that had ever smoked increased with age from 14.7% in the 12–13 age groups to 19.8% in the 20 year olds. Current smoking was most common in the 18–19 (5.5%) and 20 year (5.7%) age groups, and lowest in the 14–15 age groups (3.6%; figure 1). However, these figures differed between genders, in that both ever and current smoking was most common in the older boys, but tended to be more common in younger girls (figure 1). Ever use of shisha was relatively common in younger age groups in both sexes, but particularly in girls (figure 1).

### Characteristics of current smokers

Detailed characteristics of current smokers are outlined in table 3. Around a quarter of current smokers started smoking before the age of 12, and two-thirds before age 16. The most common reasons given for starting smoking were stress relief (21.9%) and peer pressure (20.0%). Most smokers obtained cigarettes by purchase from shops, and under half reported any difficulty doing so. Age was not a common barrier to purchase. Over half reported a regular cigarette brand (the most popular being *Bond Street*), and a third spent >D40 ($0.9) on cigarettes per day. Of the 4.4% current smokers, 13.2% had smoked in all 30 days preceding the survey, and over half had smoked two or more cigarettes per day on the days that they smoked. One in four had also used smokeless tobacco in the past 30 days. More than half (55.6%) of current smokers reported wanting to stop smoking and having tried to quit in the last 12 months (54. 5%), but only a quarter had received advice or help to quit or used nicotine replacement therapy (NRT) to help them stop smoking.

**Table 3:** Smoking characteristics of current smokers

Characteristics*	Categories	Total n=455, n (%)
Age of initiation of smoking	≤7 years old	34 (8.0)
	8–9 years	20 (4.7)
	10–11 years	42 (9.9)
	12–13 years	77 (18.2)
	14–15 years	109 (25.8)
	≥16 years	140 (33.1)
The reason for initiation of smoking cigarettes	Peer pressure	83 (20.0)
	Loneliness	73 (17.5)
	Family influence	63 (15.1)
	Curiosity	69 (16.6)
	Stress relief	91 (21.9)
	Others	36 (8.6)
Factors that most influence participant to smoke	Friends and family smoking	83 (19.9)
	The taste and feeling of it	144 (34.8)
	To relief stress	97 (23.2)
	All of the above	37 (8.8)
	Do not know	56 (13.4)
Source of cigarettes	Corner shop	161 (39.1)
	Street vendor	51 (12.4)
	From someone else	76 (18.4)
	Other ways	123 (29.9)
Refuses to sell cigarette because of participant’s age	Did not try to buy during the past 30 days	128 (31.0)
	Yes	83 (20.1)
	No	210 (48.7)
Brands of cigarettes used	No usual brand	181 (43.9)
	Piccadilly	30 (7.2)
	Monte Carlo	37 (8.9)
	Bond Street	103 (25.0)
	Business Royal	37 (8.9)
	Benson & Hedges	7 (1.7)
	Marlboro	6 (1.4)
	Others	11 (2.6)
How easy or difficult would it be to get cigarettes	Very difficult	86 (20.8)
	Fairly difficult	45 (10.9)
	Fairly easy	52 (12.5)
	Very easy	163 (39.4)
	I do not know	67 (16.2)
Amount spent on cigarettes/day	≤D10	161 (38.8)
	D11–D20	68 (16.3)
	D21–D40	52 (12.5)
	D41–D60	109 (26.2)
	D61–D80	5 (1.2)
	>D80	20 (4.8)
Number of days smoked during the past 30 days	1–2 days	147 (35.2)
	3–5 days	93 (22.3)
	6–9 days	53 (12.7)
	10–19 days	52 (12.4)
	20–29 days	17 (4.0)
	All 30 days	55 (13.1)
Number of cigarettes smoked during the past 30 days	<1 cigarette/day	58 (13.9)
	1 cigarette day	138 (33.1)
	2–5 cigarettes/day	132 (31.7)
	6–10 cigarettes/day	45 (10.8)
	11–20 cigarettes/day	21 (5.0)
	>20 cigarettes/day	22 (5.2)
Type of tobacco used†	Cigarettes	412 (90.5)
	Hand-rolled cigarettes	212 (46.5)
	Pipes	158 (34.7)
	Cigars	175 (38.4)
	Smokeless tobacco	113 (24.8)
Where participant mostly smokes	At school	27 (6.5)
	At home	82 (19.8)
	At a friend’s house	149 (36.0)
	At a street corner	64 (15.5)
	Others	91 (22.0)
Who the participant smokes with most of the time	Alone	67 (16.5)
	With friends	249 (61.3)
	With brothers/sisters	19 (4.6)
	Others	71 (17.4)
Want to stop smoking	Yes	228 (55.6)
	No	71 (17.3)
	I do not know	111 (27.0)
Received NRT	Yes	94 (22.9)
	No	316 (77.0)

*Some missing values.

†In addition to cigarette could respond to any.

NRT, nicotine replacement therapy.

### Factors associated with current smoking and ever use of shisha

The associations between current smoking (cigarettes, cigars and pipes) and of ever use of shisha and sociodemographic characteristics are outlined in table 4. After adjustment for age, gender and urban or rural location, current smoking was less common among girls (OR 0.24, 95% CI 0.19 to 0.31) and more common among students attending private schools (OR 1.69, 95% CI 1.29 to 2.22), of Christian (OR 1.56, 95% CI 1.09 to 2.24) or other faiths (OR 3.17, 95% CI 1.73 to 5.82) compared with Muslims, living with parents (OR 1.39, 95% CI 1.06 to 1.81), had smoking allowed in their homes (OR 1.67, 95% CI 1.30 to 2.13), with family members who smoked or had one or more friends who smoked. Ever use of shisha was more common in older age groups, and the gender difference less marked than for smoking (OR for girls 0.52, 95% CI 0.44 to 0.61). In other respects, risk factors for shisha use were similar to those for ever smokers.

**Table 4:** Prevalence and determinants of smoking among current smokers and ever shisha smokers in the study population

Characteristic	n =10 289	Current smokers n=455 (%)	Adjusted OR (95% CI)	P value*	Ever shisha users n=834 (%)	Adjusted OR (95% CI)	P value*
Age group (years)							**0.031**
12–14	2256	89 (3.9)	1	**0.0003**	149 (6.6)	1	
15–17	5284	213 (4.0)	1.02 (0.79 to 1.31)		440 (8.3)	1.25 (1.02 to 1.53)	
18–20	2746	153 (5.5)	1.43 (1.09 to 1.87)		245 (8.9)	1.21 (0.96 to 1.520	
Gender				**<0.001**			**<0.001**
Boys	4567	364 (7.9)	1		523 (11.4)	1	
Girls	5722	91 (1.5)	0.24 (0.19 to 0.31)		311 (5.4)	0.52 (0.44 to 0.61)	
School locality				0.182			0.331
Rural	2453	118 (4.8)	1		138 (5.6)	1	
Urban	7833	337 (4.3)	0.84 (0.66 to 1.08)		696 (8.8)	1.11 (0.89 to 1.37)	
School type				0.257			0.100
SSS	5785	205 (3.5)	1		417 (7.2)	1	
UBS	4504	250 (5.5)	1.14 (0.90 to 1.46)		417 (9.2)	0.85 (0.71 to 1.02)	
School funding				<0.001			**<0.001**
Public	7678	320 (4.1)	1		449 (5.8)	1	
Grand–aided	1052	37 (3.5)	0.91 (0.62 to 1.32)		60 (5.7)	0.94 (0.70 to 1.26)	
Private	1559	98 (6.2)	1.69 (1.29 to 2.22)		325 (20.8)	4.39 (3.66 to 5.25)	
Religion				**<0.001**			**<0.001**
Muslim	9564	398 (4.1)	1		742 (7.7)	1	
Christian	602	41 (6.8)	1.56 (1.09 to 2.24)		66 (10.9)	1.12 (0.84 to 1.49)	
Other	103	15 (14.5)	3.17 (1.73 to 5.82)		20 (19.4)	2.86 (1.68 to 4.87)	
Living with parents				**0.014**			**0.002**
No	2029	76 (3.7)	1		127 (6.2)	1	
Yes	8250	377 (4.5)	1.39 (1.06 to 1.81)		706 (8.5)	1.38 (1.12 to 1.69)	
Home smoking				**<0.001**			**0.016**
No	7295	249 (3.4)	1		551 (7.0)	1	
Sometimes	1085	75 (6.9)	1.82 (1.36 to 2.43)		110 (10.1)	1.34 (1.06 to 1.69)	
Yes	1906	131 (6.8)	1.67 (1.30 to 2.13)		173 (9.0)	1.23 (1.00 to 1.50)	
Family smoking				**<0.001**			**<0.001**
None	7364	236 (3.2)	1		513 (6.9)	1	
Mother	274	30 (10.9)	2.58 (1.64 to 4.09)		38 (13.8)	2.14 (1.45 to 3.16)	
Father	1199	89 (7.4)	1.52 (1.15 to 2.01)		124 (10.3)	1.34 (1.04 to 1.65)	
Sibling	718	66 (9.1)	1.68 (1.23 to 2.29)		87 (12.1)	1.42 (1.09 to 1.85)	
Others	729	34 (4.6)	0.97 (0.65 to 1.43)		72 (9.8)	1.16 (0.87 to 1.53)	
Friends who smoke				**<0.001**			**<0.001**
None	6790	147 (2.1)	1		368 (5.4)	1	
One	673	52 (7.7)	2.48 (1.75 to 3.50)		72 (10.6)	1.77 (1.33 to 2.36)	
Two	356	46 (12.9)	4.10 (2.82 to 5.96)		52 (14.6)	2.47 (1.77 to 3.44)	
Three or more	762	144 (18.8)	5.92 (4.54 to 7.72)		177 (23.2)	3.55 (2.84 to 4.43)	
Not sure	1699	66 (3.8)	1.58 (1.16 to 2.14)		164 (9.6)	1.54 (1.26 to 1.88)	

*Wald’s P value adjusted for age, gender and rural or urban schools.

Bold typeface indicates significance.

SSS, Senior Secondary Schools; UBS, Upper Basic Schools.

## Discussion

This is the first study to provide detailed data on smoking and other forms of tobacco use in a nationally representative sample of adolescent school students in The Gambia. We found that around 1 in 4 boys and 1 in 10 girls had ever tried smoking, but that smoking within the past 30 days was relatively uncommon, especially in girls. Young people in our sample were more likely to smoke if family or friends smoked and if smoking was allowed in the home, and generally found it easy to access cigarettes. Smoking was more common among privately educated students and among those who were not Muslim. Most smokers wanted to quit. As a result of anecdotal reports during the piloting of our study, we also uncovered significant experimentation with shisha smoking, for which the gender gap in prevalence was much less marked than for cigarette, cigar or pipe smoking. Smoking was also most common in the older boys, but tended to be higher in younger girls than older girls.

Our study has some limitations. First for logistical reasons we used a self-administered questionnaire; students may have under-reported or over-reported their answers. Nevertheless, several studies have reported high reliability of the results on self- administered teenage smoking questionnaires.^14 15^ Second, the survey was limited to students. It may not represent the smoking prevalence of all youths aged 12–20 years in The Gambia. However, based on data from the Ministry of Education, the gross enrolment rates are relatively high with 68.12% and 41.2% for UBS and SSS, respectively.^16^ We also had relatively higher proportion of girls in our sample than boys, which was slightly more marked than the national male: female enrolment ratio of 48:51^17^; we recognise that boys may have been under-represented as a result of taking employment or migrating out of the country.

Despite some limitations, our study has a number of strengths. The participation rate among those sampled was extremely high and the sample is highly representative of the total population in this age group. Forty-five per cent of the population are aged ≤15 years and about 31% are aged between 16 and 19 years.^12^ Furthermore, 100% of invited schools participated; UBS and SSS schools were sample from schools throughout the country. Moreover, results from this study are likely to reflect the situation in other sub-Saharan African countries at a similar stage of the tobacco epidemic. Furthermore, the findings from this study indicate the need to assess patterns of shisha smoking in other parts of sub-Saharan Africa.

Previous studies of smoking among students in The Gambia are limited, the most recent and widely quoted being the 2008 Global Youth Tobacco Survey (GYTS) survey. This study estimated a slightly higher overall prevalence of smoking than in ours, but this could well reflect the restricted local sampling frame used in GYTS.^11^ The low prevalence of conventional smoking among girls is consistent with earlier studies in The Gambia^18–20^ and in other LMICs in Africa and elsewhere.^15 21–23^ This can be attributed to the fact that in many African social cultures, smoking is generally more acceptable in men than in women.^24 25^ Our findings on shisha use were surprising however. Shisha smoking is known to be more common in Arab populations, and studies have shown dramatic increases in shisha smoking particularly among young people in the Middle East.^7 26^ To our knowledge this is the first study to report shisha smoking in The Gambia, and in conjunction with findings elsewhere^27–29^ indicate that shisha use may evolve into a significant health problem in such countries, particularly since a high proportion of shisha smokers does not consider shisha to be a tobacco product. Over 70% of ever shisha users in our study reported themselves to be never smokers of tobacco.

Our finding that current and ever shisha smokers were more likely to have parents and friends who smoke, have smoking allowed in their homes and to be attending a private school is consistent with previous studies. Private school attendance is a marker of relative wealth, making tobacco more affordable, and smoking among the relatively advantaged is a typical pattern of the early stages of uptake of smoking in many developing countries.^30^ Our finding that Muslims were particularly unlikely to smoke is also consistent with evidence from Ghana,^22^ and with wider evidence on smoking and spirituality.^31–33^ The Gambia is a highly religious country with predominantly Muslims (95%) and Christians (4%), thus cultural factors may have a strong influence on smoking prevalence. Furthermore, understanding cultural factors is essential in understanding the pattern of tobacco use among young people. In addition, cultural differences has been showed to be associated with tobacco smoking and suicidal ideation among 12–15 year old school-age children.^34^

Our results show that smoking imposes a significant economic cost on young smokers, with over 30% spending >D40 ($0.9) on smoking per day. Given the fact that about 50% of Gambians lived below the poverty line of $1.25/day in 2010,^35^ smoking is likely to exacerbate poverty at both individual and national level.^36 37^ Our finding that access to tobacco products was easy for our respondents, regardless of age, is common in African countries: in Cote d’Ivoire and South Africa, 68.9% and 68.7%, respectively, of students who smoke cigarettes were not refused the sale of cigarettes because of their age.^38^ Despite relatively advanced tobacco control legislation in The Gambia, with the 1998 Prohibition of Public Smoking Act, the 2003 Tobacco Product Advertisement Bill and the ratification of the WHO Framework Convention on Tobacco Control (FCTC) in 2007, enacted legislation on age restriction of tobacco products purchase was not available until 2016. Although most smokers wanted to stop smoking, there are no comprehensive cessation programmes available in the public health service system in The Gambia. This lack of stop smoking support services is a common problem in many LMICs and has been reported previously.^39 40^ Developing cessation programmes that are integrated into national health and education systems in accordance with the Article 14 of the WHO FCTC^41 42^ can potentially help reduce current smoking levels.

## Conclusion

Our finding thus demonstrates data consistent with the early stages of epidemic smoking in The Gambia and raises concerns that shisha smoking may be more important, particularly among girls, than might previously have been recognised. Urgent action in the form of tobacco control measures, including regular monitoring of uptake of all forms of tobacco smoking and the development of gender-specific and age-specific interventions, is required to reduce current levels and minimise uptake in the future. Further work is required to determine whether this is a problem local to The Gambia or reflects a wider pattern of tobacco use in sub-Saharan Africa.
